# Photo-activated charge transport and exciton dynamics in Cs₂NaInCl₆ double perovskite

**DOI:** 10.1038/s41598-026-55947-2

**Published:** 2026-06-04

**Authors:** Fahad K. Alshammari, Abdullah A. Alatawi, Achref Jebnouni, Amjad Salamah Aljaloud, Turki Alkathiri, Ahmed F. Almutairi, Bandar F. Alsaleh, Mohamed Bouzidi, Mohamed Ben Bechir

**Affiliations:** 1 Department of Physics, College of Science, University of Ha’il, P.O. Box 2440, Ha’il, Saudi Arabia; 2 King Abdulaziz City for Science and Technology, P.O. Box 6086, Riyadh, 11442 Saudi Arabia; 3 Medical Surgical Nursing Department, College of Nursing, University of Ha’il, Ha’il, Saudi Arabia; 4 Department of Electrical Engineering, College of Engineering, Al-Baha University, P.O. Box 65799, Albaha, Saudi Arabia; 5 Department of Mechanical Engineering, College of Engineering at Yanbu, Taibah University, Yanbu Al-Bahr, 41911 Saudi Arabia; 6 Laboratory of Spectroscopic and Optical Characterization of Materials (LaSCOM), Faculty of Sciences, University of Sfax, BP1171, 3000 Sfax, Tunisia; 7 Department of Physics, Faculty of Sciences of Gafsa, University of Gafsa, 2112 Gafsa, Tunisia; 8 Energy, Industry, and Advanced Technologies Research Center, Taibah University, Madinah, Saudi Arabia

**Keywords:** Lead-free double perovskite, Self-trapped excitons (STEs), Exciton–phonon coupling, Photoluminescence dynamics, Wide bandgap semiconductor, AC and DC transport, Materials science, Optics and photonics, Physics

## Abstract

On account of their environmentally-benign composition and structural stability, lead-free halide double perovskites have drawn increasing attention. Even though the charge-transport behavior of Cs₂NaInCl₆, one of these perovskites, remains poorly understood under illumination, it shows strong self-trapped exciton (STE) emission. Through a combined optical and electrical approach, this work investigates the interplay between electrical transport and exciton dynamics. With a wide bandgap and intrinsic STE-mediated blue emission, a highly crystalline cubic phase is confirmed by the optical and structural analyses. Multi-timescale photoinduced relaxation dynamics associated with recombination processes, lattice relaxation and exciton localization are inferred primarily from TRPL measurements and qualitatively supported by complementary transient absorption observations. Current–voltage measurements, dielectric analysis and complementary impedance spectroscopy evidence that illumination significantly reduces bulk resistance, enhances dielectric polarization and induces persistent photoconductivity. While the optical response is governed by STEs, the results indicate that photo-generated carriers dominate charge transport with an indirect influence of exciton dynamics through trap-state modulation. New insight into the optoelectronic functionality of lead-free double perovskites is provided through a physically-consistent framework proposed to correlate macroscopic transport properties with microscopic excitonic behavior.

**Supplementary Information:**

The online version contains supplementary material available at 10.1038/s41598-026-55947-2.

Owing to the remarkable tunability of their electronic structure, their environmental compatibility and their chemical stability, lead-free halide double perovskites with general formula A₂B⁺B³⁺X₆ stand out as wide bandgap semiconductors^1–3^. By alternating [B⁺X₆]⁵⁻ and [B³⁺X₆]³⁻ corner-sharing octahedra, the three-dimensional and highly-ordered lattice of these perovskites provides a defect-tolerant and structurally-rigid framework able to corroborate unconventional photo-physical phenomena, long-lived electronic states and strong excitonic effects^4,5^. Accordingly, In-based chloride double perovskites have drawn attention on account of the resulting formation of strongly confined STEs controlling not only their emission behavior but also their absorption, the strong ionicity of the In–Cl bond and the parity-forbidden nature of the valence-to-conduction transition^6,7^.

Cs₂NaInCl₆ has emerged as a remarkably unique system compared to others. Its deep-UV bandgap and intrinsically excitonic nature has made it a distinguished model for exploring relaxation dynamics and STE formation in lead-free perovskites, whereas its highly symmetric $\:Fm\stackrel{-}{3}m$ structure shows a noticeable structural and thermal robustness^8–10^. Developments have lately demonstrated that subtle modifications of the B-site chemistry (for example Ag substitution) and the incorporation of lanthanide dopants deeply modify the optical response of Cs₂NaInCl₆, which allows near-infrared emission through unconventional In–Cl → Ln³⁺ transfer processes, strong charge-transfer transitions and efficient sensitization pathways^11–15^. Consequently, the central role of lattice–exciton coupling that determines the optical properties of this material and the strong sensitivity of the excitonic landscape to local octahedral distortions are confirmed.

The charge-transport properties of Cs₂NaInCl₆ are still unknown to a fairly large degree in spite of recent advances^11–15^. Exciton-related processes, dopant sensitization and photoluminescence were the center of attention in prior research but no significant focus has been devoted to the electrical response such as DC photoconductivity, electric modulus, AC conductivity, dielectric relaxation and impedance. The direct correlation between electrical transport properties and exciton dynamics remains largely unexplored while doping-induced luminescence enhancement and STE-mediated optical emission have been the main focus of prior studies^11–15^. However, in order to assess the suitability of Cs₂NaInCl₆ for radiation-detection, scintillation, dielectric and optoelectronic applications, it is vital to understand how illumination changes relaxation dynamics, space-charge polarization, carrier mobility and trap filling. Correlating optical signatures including lattice relaxation and STE formation with such electrical phenomena as persistent photoconductivity, non-Debye relaxation, dielectric dispersion and resistance evolution is fundamental to building a unified physical description of the material, taking into account the strong exciton–phonon interaction which is typical of In-based perovskites.

This paper provides the first thorough multi-scale study of Cs₂NaInCl₆ integrating electrical measurements that incorporate AC (impedance spectroscopy) and DC (I–V photoconductivity) regimes, optical spectroscopy (including transient absorption as a complementary probe, TRPL, temperature-dependent PL and steady-state PL) and also structural characterization. Particularly, a direct comparison between charge-transport mechanisms and excitonic processes is allowed through the combination of electrical characterization with optical spectroscopy, which enables this work to go beyond prior studies. This combined approach enables a qualitative investigation of the interplay between long-lived photocarriers, trap-state populations and localized excitonic processes. Such multi-timescale relaxation dynamics are commonly associated with carrier trapping, lattice relaxation and localized excitonic processes in halide double perovskites^16,17^. The transient absorption results are used in the present work so as to qualitatively support these dynamics. The combined optical and electrical results suggest that photoexcitation alters the carrier landscape of Cs₂NaInCl₆, which indicates a coherent framework in which STEs not only indirectly influence transport via carrier stabilization and recombination dynamics but also dominate the optical response while the electrical transport is governed by trap filling and space-charge formation through mobile charge carriers. Therefore, Cs₂NaInCl₆ can be observed as an application-relevant, intrinsically excitonic and robust lead-free material for radiation-detection technologies, dielectric components and also future ultraviolet photonics. It is crucial to distinguish between these two roles: photo-generated carriers interacting with trap states primarily dominate long-range charge transport while STEs are responsible for radiative recombination processes.

## Experimental section

### Materials

Cesium chloride (Sigma-Aldrich, 99.9%), Sodium chloride (Sigma-Aldrich, $\:\ge\:$ 99.0%), Indium(III) acetate (Sigma-Aldrich, 99.99% trace metals basis), 2-Propanol (Sigma-Aldrich, ≥ 99.0%, meets USP testing specifications, USP/NF) and Hydrochloric acid (Sigma-Aldrich, 37%, for analysis max. 0.001 pp M Hg EMSURE^®^) were employed precisely as provided without conducting any additional purification processes.

### Synthesis

Following a one-pot hydrothermal method taken from standard procedures and used for halide double perovskites, high-quality Cs₂NaInCl₆ double perovskite crystals were synthesized. Indeed, 1.6 mmol of CsCl, 0.8 mmol of NaCl and 0.8 mmol of Indium(III) acetate were typically dissolved in 8 mL of concentrated HCl (10 M) inside a 20 mL Teflon-lined stainless-steel autoclave. In order to ensure a homogeneous precursor solution, the mixture was stirred until complete dissolution. Then, the sealed autoclave was heated at 180 °C for 12 h so as to promote crystal growth under hydrothermal conditions. The system was later cooled slowly to 30 °C at a controlled rate of 3 °C h⁻¹ to facilitate the formation of well-defined crystals which were collected by filtration, washed three times with isopropyl alcohol to remove residual acid and surface impurities and then dried at 60 °C.

### Device characterization

The crystal structure of Cs₂NaInCl₆ was characterized by powder X-ray diffraction by means of a Rigaku MiniFlex 600 diffractometer equipped with a Cu Kα source (λ = 1.5406 Å). Diffraction patterns were recorded at room temperature over the 2θ range 10–50° with a step size of 0.05°. To confirm the $\:Fm\stackrel{-}{3}m$ cubic symmetry, the data were refined using the CELREF3 program. By means of a TGA-9000 system, thermal stability was evaluated by thermogravimetric analysis under nitrogen flow. Almost 12.3 mg of the sample was heated from room temperature to 800 °C at 5 °C·min⁻¹. TGA and DTG profiles were collected.

Using a Shimadzu UV-2550 spectrophotometer, optical absorption spectra were measured at room temperature. Steady-state photoluminescence (PL) spectra were obtained under 318 nm excitation by means of a Shimadzu RF-1501 spectrofluorometer. PL measurements were performed in the dark and under continuous illumination. Temperature-dependent PL (80–300 K) was recorded by a closed-cycle cryostat. Time-resolved PL (TRPL) decays were measured using a FLOROCUBE system equipped with a NanoLED pulsed source operating at 365 nm, while the emitted signal was collected through a TBX-04-D detector. Decays were measured at ~ 445 nm and corrected for the instrument response function.

Ultrafast transient absorption (TAS) measurements were done with an amplified Ti: sapphire laser system (800 nm, ~ 100 fs, 1 kHz). Using an optical parametric amplifier, pump pulses were generated at 325 nm. A broadband white-light probe continuum was produced in a sapphire plate. A motorized delay stage offered a femtosecond–nanosecond time resolution, whereas a dual-beam detection scheme was used so as to enhance spectral stability. TAS measurements were conducted at 300, 150 and 80 K. Through a multi-exponential model, the kinetic traces were analyzed near 445 nm.

Electrical characterization was conducted by means of current–voltage (I–V) measurements and impedance spectroscopy (IS). The crystals were ground and pressed into pellets of 0.96 mm thickness and 8 mm diameter with gold electrodes sputtered on both faces. Using a 0.5 V AC amplitude, IS measurements were carried out with a Zahner Zennium workstation over the 0.1 Hz–10 MHz frequency range at room temperature. Dark measurements were collected in a sealed light-tight chamber. However, illuminated measurements employed a 365 nm UV-LED source (≈ 10 mW cm⁻²). After illumination times of 0, 15, 60 and 120 min, Spectra were acquired and analyzed using ZView. DC I–V curves were recorded between 0 and 10 V under the same dark and illuminated conditions by means of a Keithley 2400 SourceMeter.

### Results and discussion

**Fig. 1:**
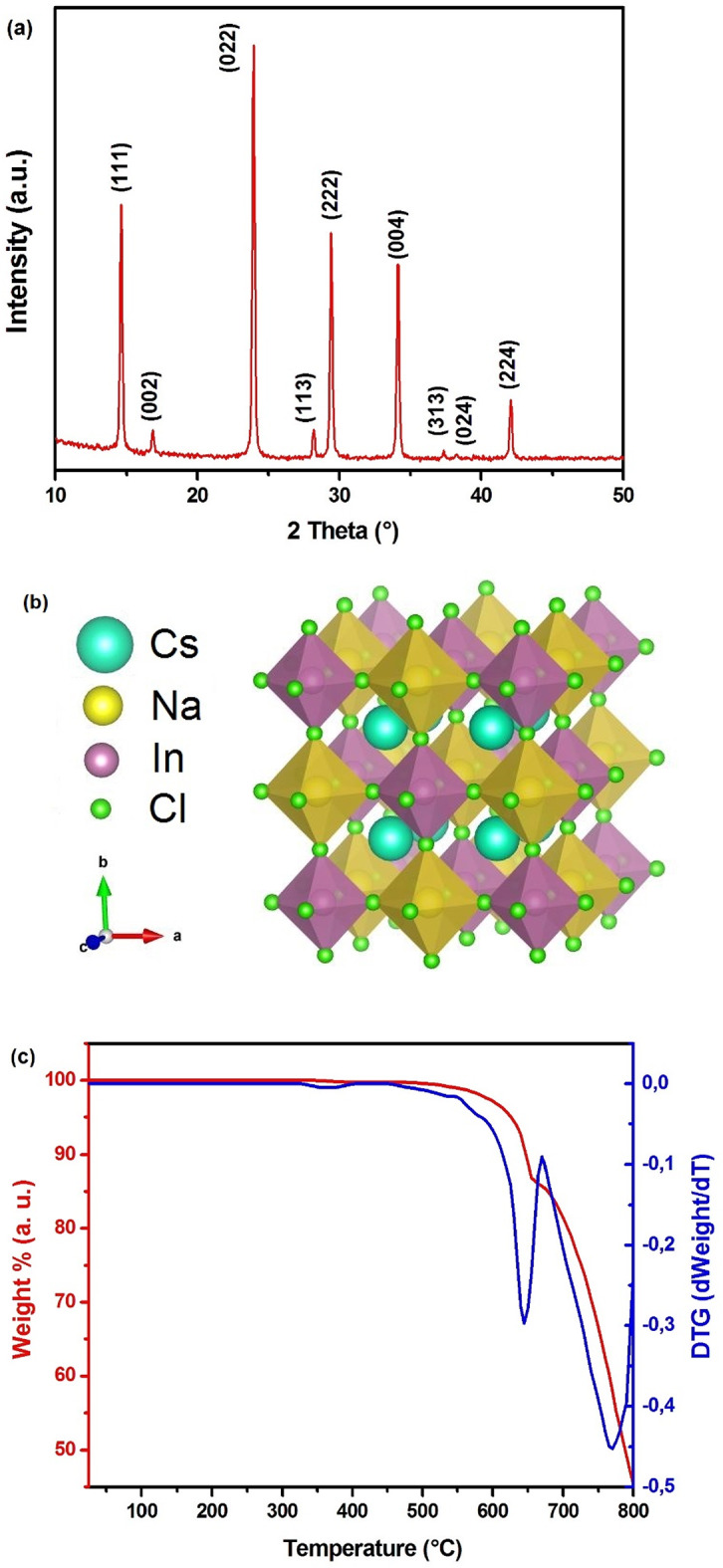
(**a**) X-ray diffraction pattern of Cs₂NaInCl₆, (**b**) Crystal structure showing NaCl₆ and InCl₆ octahedra, and (**c**) Thermogravimetric analysis (TGA) curve.

Figure 1a shows the powder X-ray diffraction pattern of Cs₂NaInCl₆, confirming sharp and well-defined reflections, which demonstrates that the compound crystallizes as a single highly-ordered phase. There are no secondary peaks detected, indicating the absence of impurity phases. All reflections are possibly indexed in a cubic double-perovskite structure assigned to the $\:Fm\stackrel{-}{3}m$ space group, which is in agreement with data previously reported in the literature^15,18^.

Table 1 displays the diffraction data refined by means of CELREF3, which produces a = b = c = 10.5010(12) Å and a unit-cell volume of 1157.97(13) Å³. With a maximum deviation smaller than 0.05° in 2θ, the calculated and experimental diffraction lines match closely. The refinement residual (√Σ(2θₒ–c)² / (N_ref_ – N_par_) = 0.0256) is very low, which confirms an excellent fit between the observed and simulated data. Fig. S1 illustrates the refinement profile obtained from CELREF3 which highlights this good agreement.

Cs₂NaInCl₆ structurally adopts the typical arrangement of ordered double perovskites. As shown in Fig. 1b, each Na⁺ cation is surrounded by six Cl⁻ anions forming [NaCl₆]⁵⁻ octahedra, which alternate with [InCl₆]³⁻ octahedra throughout the crystal framework^19^. These octahedra are corner-sharing, resulting in a rigid and symmetrical three-dimensional network. The refined lattice parameter (10.501 Å) is slightly smaller than that of Cs₂NaEuCl₆ (10.7478 Å), which is in agreement with the smaller ionic radius of In³⁺ (0.80 Å) compared with Eu³⁺ (0.95 Å). While maintaining the overall cubic symmetry, this size reduction generates a more compact lattice, which is consistent with the Goldschmidt tolerance-factor tendency observed for A₂B⁺B³⁺X₆ perovskites.

**Table 1:** X-ray diffraction data for Cs₂NaInCl₆, including lattice parameters and unit cell volume.

Parameter	Symbol	Value	Standard error	Remarks
Lattice constant	a = b = c	10.5010 Å	± 0.0118 Å	Cubic cell
Cell volume	V	1157.97 Å³	± 1.30 Å³	–
Space group	–	$\:Fm\stackrel{-}{3}m$	–	Double-perovskite symmetry
Radiation wavelength	λ	1.5406 Å (Cu Kα)	–	–
Zero shift	–	0.000°	–	Perfect alignment
Refinement residual	√Σ(2θₒ–c)² / (Nref – Npar)	0.0256	–	Excellent fit
Average 2θ deviation	Δ2θ	< 0.05°	–	High indexing precision
Refinement software	–	CELREF3 (v3)	–	–

In order to examine the thermal behavior of the material, thermogravimetric (TGA) and derivative thermogravimetric (DTG) analyses were carried out as illustrated in Fig. 1c. The TGA curve remains in essence flat up to almost 620 °C, which confirms that Cs₂NaInCl₆ is thermally stable within this range. A small mass loss associated with a single and broad DTG peak appears near 640 °C, suggesting a limited decomposition process probably because of chloride sublimation^20^. Overall, the results demonstrate that Cs₂NaInCl₆ is a thermally robust double perovskite which is not only stable under high temperatures but also comparable in durability to related compounds such as Cs₂NaBiCl₆ and Cs₂NaEuCl₆^4,16^. This stability supports its suitability for future photonic and optoelectronic applications to a great degree.

**Fig. 2:**
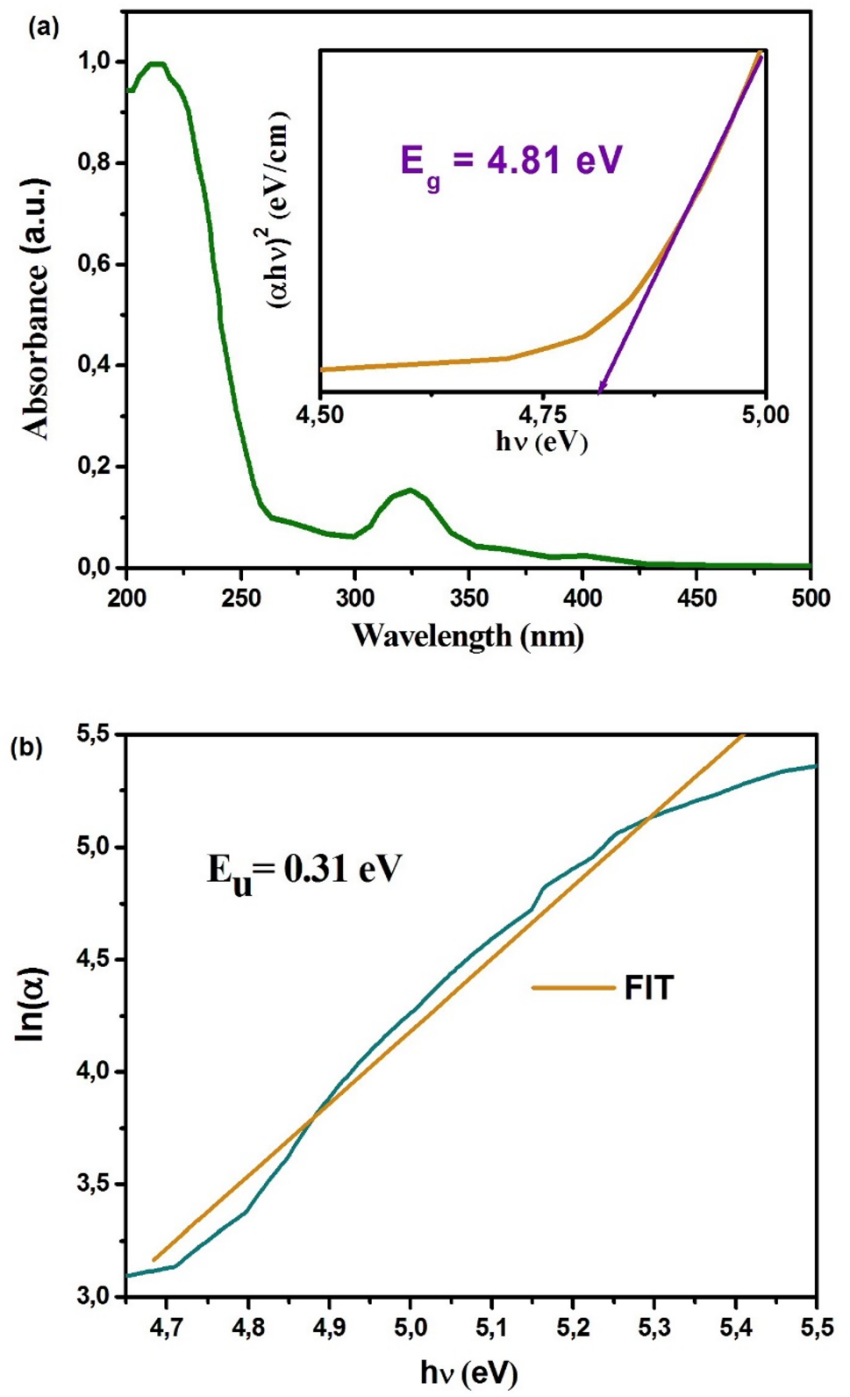
(**a**) Optical absorption spectrum of Cs₂NaInCl₆, and (**b**) Urbach energy plot.

The optical absorption spectrum of Cs₂NaInCl₆ recorded at room temperature is shown in Fig. 2a. The compound indicates a strong absorption edge in the ultraviolet region (200–400 nm), which is a characteristic of a wide bandgap semiconductor. As illustrated in the inset of Fig. 2a, the optical bandgap (E_g_) was estimated from the Tauc plot, assuming a direct electronic transition. The presence of parity-forbidden transitions causes a weak absorption edge even though Cs₂NaInCl₆ is commonly described as a direct bandgap semiconductor^15,19^. Being widely reported, this behavior is associated with symmetry-induced restrictions on optical transitions which can be partially relaxed through chemical or structural modifications^14^. As commonly reported for similar In-based halide double perovskites, the extracted bandgap should thus be considered as an effective optical bandgap^14^. The corresponding Tauc relation is expressed as follows in Eq. (1):

1 $$\left( {\alpha hv} \right)^{{{\mathrm{1}}/{\mathrm{n}}}} = \beta \left( {hv - E_{g} } \right)$$

where α represents the absorption coefficient, hν is the photon energy, β stands for a proportionality constant, and n refers to an exponent that is equivalent to ½ for direct allowed transitions.

The direct optical bandgap of Cs₂NaInCl₆ proved to be 4.81 eV by extrapolating the linear portion of the (αhν)² vs. hν curve to the energy axis. This relatively high value affirming the insulating behavior of the material is in line with values reported for similar In-based halide double perovskites in the literature (4.3–4.8 eV)^12,15,20^. The wide gap originates not only from the parity-forbidden Cl–p → In–s transition which shifts the absorption edge towards the deep-UV region but also from the strong In–Cl ionic bonds.

The Urbach energy (E_u_) gives insight into the degree of structural disorder and defect-induced localized states near the band edges. The relationship between the absorption coefficient and photon energy near the absorption tail follows Urbach’s law as expressed in Eq. (2):

2 $$a = a_{0} \,\exp \left( {\frac{{hv}}{{E_{u} }}} \right)$$

which can be linearized by taking the natural logarithm as follows in Eq. (3) :

3 $$\:\mathrm{l}\mathrm{n}\:\left(\alpha\:\right)=\mathrm{ln}\left(\:{\alpha\:}_{0}\right)+\:\frac{1}{{E}_{u}\:}\:\mathrm{h}{\upnu\:}$$

Figure 2b shows the plot of ln(α) as a function of hν which yields a straight line whose slope corresponds to 1/E_u_. From this slope, the Urbach energy for Cs₂NaInCl₆ was found to be 0.31 eV. This rather small value demonstrates a low density of localized states and, therefore, a low structural disorder in the crystal, which is in line with the high crystallinity proven by the XRD analysis^21–24^. The obtained E_u_ value is slightly smaller than those generally reported for doped or defect-rich halide double perovskites (0.35–0.45 eV), which indicates the structural integrity and optical purity of the synthesized material^21–24^.

**Fig. 3:**
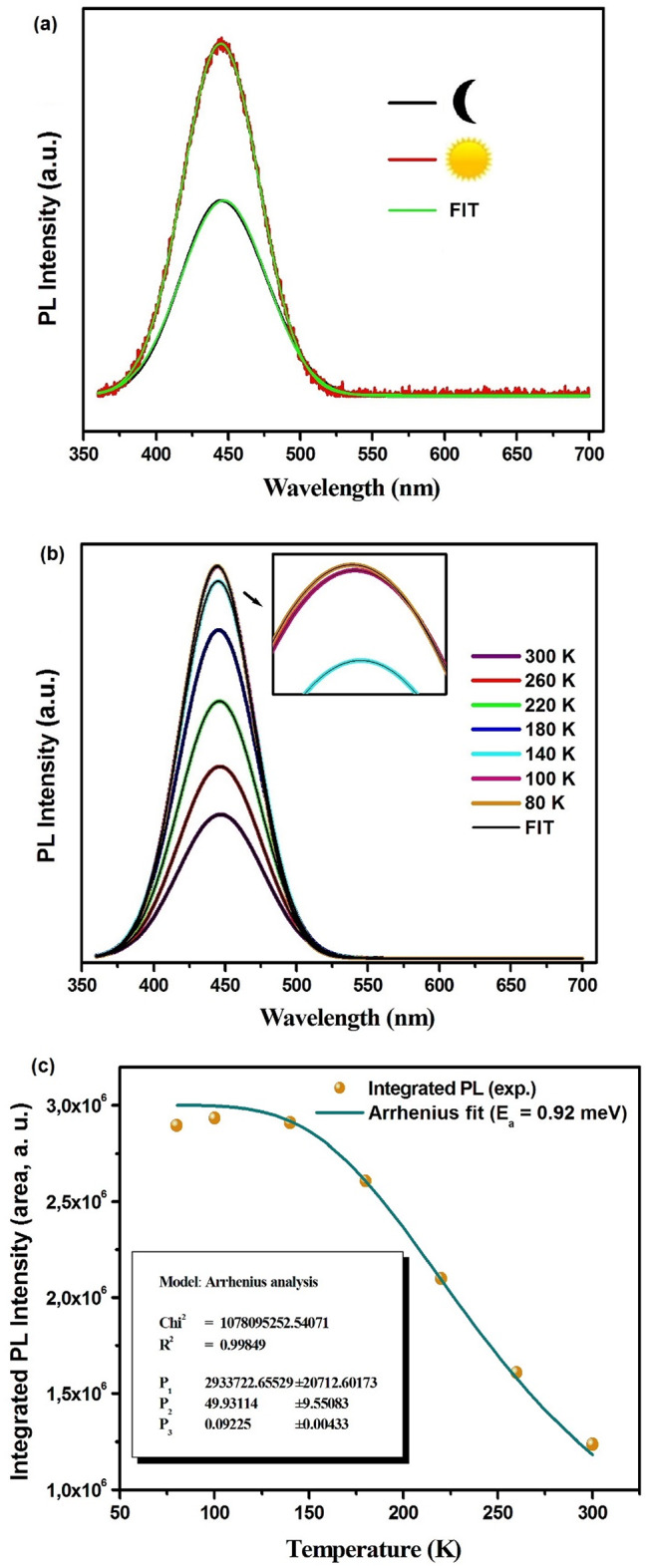
(**a**) Room-temperature PL spectra under dark and illumination conditions. (**b**) Temperature-dependent PL spectra from 80 to 300 K. (**c**) Arrhenius plot of the integrated PL intensity with the corresponding fit.

The room-temperature PL spectra of Cs₂NaInCl₆ that were recorded with a 318 nm excitation wavelength under dark and illumination conditions are displayed in Fig. 3a. Both spectra show a broad emission band in the blue region (centered near 445 nm), originating from radiative recombination of STEs within the In–Cl octahedral network^11,25,26^. Contributions from surface effects or defect-related states cannot be entirely excluded while the broad emission is characteristic of intrinsic self-trapped excitons. Nevertheless, STE-mediated recombination represents the dominant emission mechanism, which is strongly suggested not only by the spectral features but also by the low Urbach energy.

The experimental data were fitted to quantify the emission characteristics using a single Gaussian function expressed in Eq. (4)^27^:

4 $$\:I\left(\lambda\:\right)=A{e}^{\left[-\raisebox{1ex}{${\left(\lambda\:-{\lambda\:}_{0}\right)}^{2}$}\!\left/\:\!\raisebox{-1ex}{${2\sigma\:}^{2}$}\right.\right]}+C$$

where *A* is the peak amplitude, *λ₀* represents the peak wavelength and *σ* indicates the standard deviation related to the bandwidth while *C* refers to the baseline offset. The full width at half maximum (FWHM) proved to be as follows in Eq. (5):

5 $$\:FWHM=2\sqrt{2ln2}\sigma\:$$

Table 2 provides a summary of the fitted parameters obtained for both conditions. The emission maximum occurs at 447 nm with a FWHM of 70 nm under dark conditions. As soon as the sample is exposed to illumination, the intensity increases by approximately a factor of two, accompanied by a bandwidth narrowing to 63 nm and a slight blue shift to 445 nm. The integrated area of the emission also goes up, which shows an improvement in radiative recombination efficiency. It should be noted that, while the recombination probability and the carrier population are mainly increased by additional illumination without altering the emission mechanism, intrinsic STE emission has already been generated by the excitation source used for PL measurements.

**Table 2:** Fitted parameters for room-temperature photoluminescence (PL) spectra under dark and illumination conditions.

Condition	Peak (nm)	FWHM (nm)	Amplitude (counts)	Baseline (counts)	Area (counts·nm)
Dark	447	70	1.66 × 10⁴	3.99	1.24 × 10⁶
Illumination	445	63	2.99 × 10⁴	97.28	1.99 × 10⁶

An improved uniformity of the excitonic environment under continuous excitation or a decline in electron–phonon coupling strength is shown by the narrowing of the emission profile.

By and large, it is demonstrated, through the PL behavior, that a stable and intrinsic blue emission of Cs₂NaInCl₆ with photo-activation is preserved even under external illumination, which generates more efficient excitonic recombination. Such change is in agreement with the optical responses reported for wide bandgap indium-based halide double perovskites^12,20^.

Under a 318 nm excitation wavelength, the temperature-dependent PL spectra of the compound recorded between 80 and 300 K are displayed in Fig. 3b. A broad blue emission band of Cs₂NaInCl₆ is revealed. As soon as the temperature decreases, the intensity of the emission band noticeably rises, showing the typical thermal quenching behavior of the STE emission in wide bandgap halide double perovskites.

The spectral profiles were fitted by means of a single Gaussian function. The extracted parameters are summarized in Table 3. The emission maximum shifts slightly from 447 nm at 300 K to 444 nm at 80 K upon cooling, whereas the FWHM goes from 70 nm to 60 nm. The gradual narrowing of the emission band demonstrates a decrease in electron–phonon coupling and enhanced localization of excitons at low temperatures. The increase in emission intensity is connected to the suppression of thermally-activated non-radiative recombination processes, which leads to more efficient radiative transitions within the In–Cl octahedral framework. It should be noted that, although a quantitative evaluation via Huang–Rhys analysis is beyond the scope of this work, strong exciton–phonon coupling is qualitatively reflected by the observed temperature dependence of the emission bandwidth.

The integrated PL intensity (I_int_) was analyzed as a function of temperature to quantify this quenching effect according to the Arrhenius model as follows in Eq. (6)^28,29^:

6 $$\:I\left(T\right)=\frac{{I}_{0}}{1+C{e}^{\left(-\frac{{E}_{a}}{{k}_{B}T}\right)}}$$

where I_0_ stands for the maximum intensity at 0 K, C represents a constant related to the non-radiative centers, E_a_ is the activation energy and k_B_ refers to the Boltzmann constant.

The corresponding Arrhenius plot is displayed in Fig. 3c. E_a_ ≈ 0.092 eV is yielded by fitting the data with Eq. (6). The fitting procedure was verified so as to ensure consistency, whereas the Arrhenius model was carefully re-examined. Nevertheless, as the robustness of the fit may be affected by the relatively weak variation of the integrated PL intensity and the limited temperature range, the obtained value should notably be considered as an approximate estimation.

The extracted activation energy (~ 92 meV) falls within the scope of the values reported in the literature (70–100 meV) for similar halide double perovskites^15,19^. These values are not only comparable to typical STE binding energies reported for In-based systems but also typically associated with stable self-trapped excitons in halide double perovskites. This energy barrier and the thermal activation which is necessary for carriers to leave STE states for non-radiative pathways match up. It is therefore demonstrated that, at higher temperatures, non-radiative losses govern, whereas the radiative recombination of STEs prevails at low temperatures owing to the increase in PL intensity.

The thermal evolution of the PL strongly supports the excitonic character and the intrinsic optical stability of Cs₂NaInCl₆. The robustness of the STE-mediated emission process in this halide double perovskite is emphasized by the consistent activation energy, the moderate FWHM narrowing and the limited spectral shift.

**Table 3:** Temperature-dependent PL fitting results, including emission peak position and full width at half maximum (FWHM).

T (K)	Peak (nm)	FWHM (nm)	Amplitude (counts)	Baseline (counts)	Area (counts·nm)
80	444	60	45450.11	0.2	2893927.2
100	445	61	45347.92	0.2	2931303.47
140	445	63	43672.88	0.2	2907552.9
180	446	64	38046.68	0.2	2606619.9
220	446	66	29789.48	0.2	2098564.57
260	446	68	22216.31	0.2	1608058.11
300	447	70	16638.14	0.2	1236501.64

**Fig. 4:**
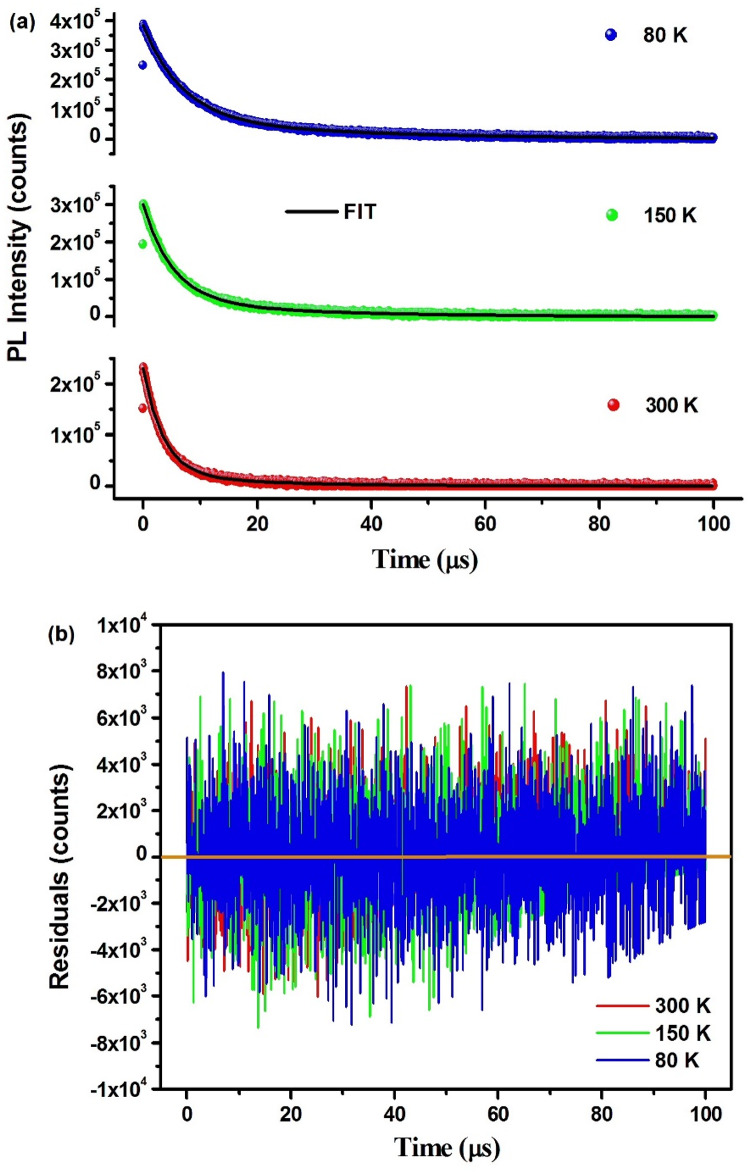
(**a**) Time-resolved photoluminescence (TRPL) decay profiles at 445 nm for various temperatures, and (**b**) TRPL fitting residuals.

In order to gain insight into the carrier recombination mechanisms and the dynamics of the emissive centers, the time-resolved photoluminescence (TRPL) behavior of Cs₂NaInCl₆ crystals was scrutinized. The decay profiles recorded at 445 nm for three representative temperatures (300, 150, and 80 K) are displayed in Fig. 4a. All curves confirm a non-single-exponential decay, which is well described by a bi-exponential model convoluted with the instrumental response function (IRF). The unique assignment of multi-exponential decay behaviors is challenging since they not only generally result from the interplay of recombination processes, lattice relaxation, exciton self-trapping and carrier trapping but are also widely observed in halide perovskites^16,17^. This fitting approach which is validated by the low and symmetric residuals shown in Fig. 4b indicates the coexistence of two distinct relaxation channels. It is important to point out that the assignment of each component to a specific physical process is not uniquely definitive, and a phenomenological description of the decay dynamics is provided by the bi-exponential fitting.

Table 4 provides a summary of the extracted lifetimes and fitting parameters. At room temperature, the decay is controlled not only by a fast component (τ₁ ≈ 3.1 µs) attributed to a rapid radiative recombination channel of self-trapped excitons (STEs) possibly involving shallow localized states but also by a slower component (τ₂ ≈ 12 µs) which probably corresponds to slower recombination pathways involving defect-assisted transitions or deeper localized states^12,15^. Both components increase steadily, reaching τ₁ ≈ 6.5 µs and τ₂ ≈ 28 µs at 80 K as soon as the temperature goes down. This gradual lifetime lengthening reveals the suppression of thermally activated non-radiative channels and the stabilization of the STEs at low temperature^11,13^. The average lifetime ⟨τ⟩ follows the same trend, developing from ~ 7 µs at 300 K to ~ 18 µs at 80 K, which is consistent with the emission improvement observed in the steady-state PL spectra^12^. Such interpretations should be treated with caution since multi-exponential fitting does not allow a unique assignment of each component but offers a phenomenological description of the recombination dynamics. Similar multi-timescale behaviors have been widely reported in halide perovskites where the observed dynamics result simultaneously from multiple processes including recombination, lattice relaxation and carrier trapping.

**Table 4:** Time-resolved photoluminescence (TRPL) fitting parameters for decay profiles at different temperatures.

T (K)	τ_1_ (µs)	τ_2_ (µs)	B_1_ (counts)	B_2_ (counts)	avg τ (µs)	*R* ^2^
80	6.46	28.48	307,523	79,949	18.22	0.9989
150	4.76	21.02	248,076	57,525	12.99	0.9979
300	3.13	12.18	195,651	39,993	7.14	0.9967

The results are predominantly consistent with STE-mediated radiative recombination across the investigated temperature range, whereas, at higher temperatures, non-radiative pathways become increasingly active. The absence of additional fast decay components and irregular oscillations indicates a low density of defect-related trap states, which is in agreement with the small Urbach energy derived from optical absorption. Such observations strongly suggest that the optical response of Cs₂NaInCl₆ is dominated by intrinsic localized excitonic recombination within the In–Cl octahedral network rather than from extrinsic impurities or surface effects. These microsecond-scale dynamics characterize wide bandgap indium halide double perovskites and underline the strong exciton–lattice interaction which dominates their photo-physical behavior. Even though the consistency with STE signatures indicates a dominant excitonic contribution, such long-lived states probably result not only from defect-related trapping processes but also from STE formation.

As shown in Supporting Information including Eq. (S1), Table S1 and Fig. S2(a–e), the TAS measurements were carried out to probe the photo-induced carrier dynamics in Cs₂NaInCl₆. The transient absorption map reveals a broad positive transient signal in the visible region that evolves over time^29–31^. Since the spectral position partially overlaps with the steady-state PL emission band centered near 445 nm, possible contributions from PL leakage, scattered emission, or other emission-related artifacts cannot be completely excluded within the present experimental configuration. Therefore, the TAS measurements are interpreted cautiously as qualitative signatures of photoinduced excited-state dynamics rather than definitive evidence of intrinsic excited-state absorption or direct STE formation.

The kinetic traces phenomenologically indicate the presence of multiple relaxation pathways exhibiting non-single-exponential decay behavior. A multi-exponential model including a slower decay extending into the nanosecond regime, an intermediate process and a fast component (tens of picoseconds) can describe the dynamics. Such timescales match post-formation relaxation processes but the initial STE formation is expected to occur on a sub-picosecond timescale beyond the resolution of the present setup. Added to the fitting equation, the detailed fitting procedure, corresponding parameters, is available in the Supporting Information.

Typically occurring on a sub-picosecond timescale, ultrafast processes such as the initial self-trapping of excitons are not directly resolved within the temporal resolution of the present measurements. Therefore, instead of the primary STE formation, the subsequent relaxation and stabilization processes of the excitonic states lead to the observed picosecond components^29–31^.

The TAS measurements suggest the presence of multi-timescale photoinduced relaxation processes that may involve carrier relaxation, lattice interactions, and exciton-related dynamics. However, due to the spectral overlap with the PL emission band and the limited temporal resolution of the experiment, these observations should be considered qualitative and phenomenological. The observed trends remain broadly consistent with the relaxation behavior identified by TRPL measurements.

**Fig. 5:**
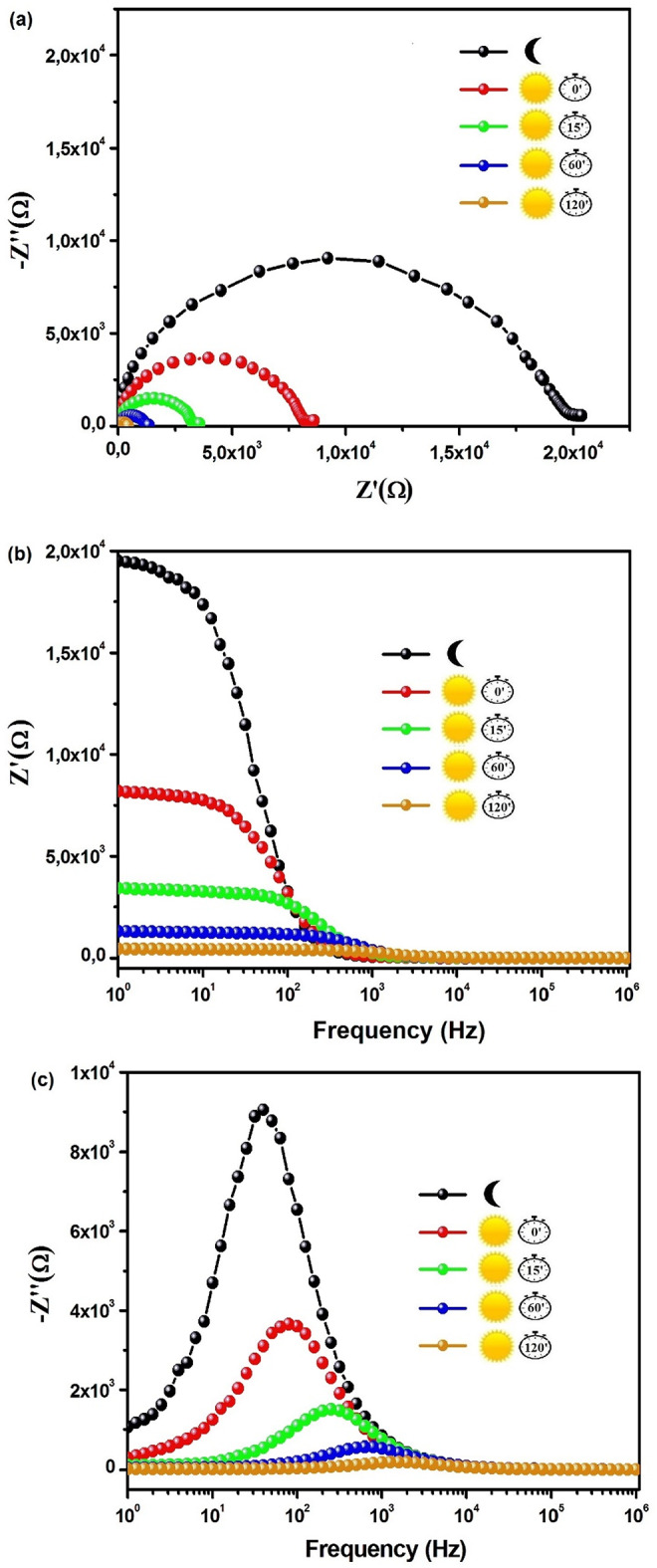
(**a**) Nyquist plot of impedance spectra for Cs₂NaInCl₆ under different illumination times, (**b**) Z’(ω) spectra showing the real part of impedance, and (**c**) –Z’’(ω) spectra showing the imaginary part and relaxation peaks.

IS was used to establish connections with the previously described optical processes and to examine the charge-transport mechanism in Cs₂NaInCl₆^32–34^. The Nyquist plots recorded after different illumination durations (0, 15, 60 and 120 min) under dark conditions are displayed in Fig. 5a. Each spectrum consists of a single depressed semicircle, which suggests that a single dominant relaxation process governs the electrical response. The absence of a second arc suggests that the grain and grain boundary contributions are not resolved separately in the explored frequency range but instead they merge into a single effective transport pathway. This behavior is in agreement with the high crystallinity, the low disorder and the narrow Urbach energy extracted from optical absorption, which altogether indicate a structurally homogeneous material. The semicircle becomes a large diameter under dark conditions, which reveals a high charge-transfer resistance^35^. A gradual and monotonic decrease in this diameter is noticed during illumination, reflecting a clear photo-induced enhancement of electrical conductivity. This evolution shows the following optical results: steady-state PL represents an increase in intensity and a slight spectral narrowing under illumination and TRPL indicates longer exciton lifetimes at lower trap densities while TAS offers additional qualitative support for the presence of long-lived photo-induced species. These optical signatures suggest that illumination modifies the carrier landscape and trap-state population of Cs₂NaInCl₆, while the optical response remains predominantly governed by localized excitonic emission. The optical and electrical measurements evolve consistently under illumination; however, the present results establish phenomenological correlations rather than a direct causal relationship between ultrafast excitonic processes and long-timescale electrical photoactivation phenomena^11–15^.

It is noteworthy that STEs do not directly contribute to long-range charge transport due to their localized nature but rather to an increase in the lifetime and population of mobile charge carriers, directly leading to a reduction in electrical resistance observed in the impedance spectra. Multiple mechanisms including surface or interface effects, defect-induced metastable states and deep trap states often bring about the origin of persistent photoconductivity in halide double perovskites. Together with the absence of electrode-dependent behavior in I–V measurements, the observed bulk-dominated impedance response in the present case suggests that, instead of surface-related phenomena, bulk trap states and their slow release dynamics primarily govern PPC. An equivalent circuit model was fitted to the data, as shown in Fig. S3, using ZView in order to quantitatively analyze the impedance response^36^. The best agreement was obtained by means of a single R₁‖CPE₁ element in series with a second CPE₂. CPE₂ accounts for the polarization taking place at the electrode–sample interfaces and also for slow space-charge accumulation at low frequencies while the parallel R₁–CPE₁ branch describes the bulk relaxation process in which contributions of grains and grain boundaries seem merged. The suitability of this model is confirmed by the excellent superposition between the simulated and the experimental spectra. As displayed in Table 5, the parameters extracted from the fits indicate a pronounced decrease in R₁ from 19.1 kΩ in the dark to 422 Ω after 120 min of illumination. This significant reduction additionally confirms the emergence of a more conductive state upon photoexcitation, which is in line with the enhanced STE emission and reduced non-radiative losses observed optically. Furthermore, the values of CPE₂ go up steadily with illumination time, which reveals a stronger interfacial polarization due to the accumulation of long-lived photocarriers. The multi-component TRPL dynamics together with the qualitative TAS observations are consistent with a non-Debye relaxation behavior associated with a broad distribution of relaxation pathways and local environments, as reflected by the exponents α < 1 exhibited by both CPE elements.

**Table 5:** Fitted parameters from impedance spectroscopy and equivalent circuit model.

	*R*_1_ (Ω)	C_e1_ (F)	α_e1_	C_e2_ (F)	α_e2_
☽	19,106	2.52E-7	0.965	8.74E-4	0.581
☀ − 0 min	7995	3.27E-7	0.962	1.82E-3	0.551
☀ − 15 min	3278	2.79E-7	0.957	3.54E-3	0.510
☀ − 60 min	1246	3.16E-7	0.947	7.79E-3	0.480
☀ − 120 min	422.2	4.60E-7	0.936	2.47E-2	0.490

As shown in Fig. 5b and c, the frequency-dependent behaviors of Z′(ω) and –Z″(ω) offer additional insight into the evolution of the charge-transport dynamics. Observed in the Nyquist representation, the high resistance is revealed through high values in the Z′(ω) spectra at low frequencies. The fact that photo-generated carriers considerably diminish the resistive component of the response is affirmed by these low-frequency values declining noticeably throughout illumination^35^. The optical evidence for the robust structural nature of the material under light exposure is in agreement with the intrinsic fast polarization processes of the lattice which are largely unaffected. This is attributed to the fact that all curves converge towards similar values at high frequencies.

Distinct relaxation peaks are revealed through the − Z″(ω) plots. With increasing illumination time, the positions of these peaks shift gradually to higher frequencies and also their amplitudes decrease. Such change matches a decline in the characteristic relaxation time (τ = 1/ω_max_), which implies that as illumination promotes carrier mobility and fills traps, charge carriers respond to the AC field more rapidly. The non-Debye behavior is characterized by the broad asymmetric shape of the peaks, corresponding to distributed relaxation pathways associated with slow recombination dynamics^37^, lattice relaxation, carrier trapping, and localized excitonic processes reflected in the TRPL measurements and qualitatively supported by the TAS observations. These impedance signatures are consistent with the presence of localized excitonic states dominating the optical response together with illumination-activated mobile carriers responsible for electrical transport.

Illumination remarkably restructures the charge-transport landscape of Cs₂NaInCl₆ as indicated by the impedance analysis. Altogether, the acceleration of relaxation dynamics, the growth of interfacial polarization, and the strong decrease in R₁ reveal that photoexcitation brings about mobile carriers and alters the excitonic environment. These effects confirm the optical findings and highlight that exciton self-trapping and trap filling play key roles in emission processes and in the electrical response of this wide bandgap indium-based double perovskite.

**Fig. 6:**
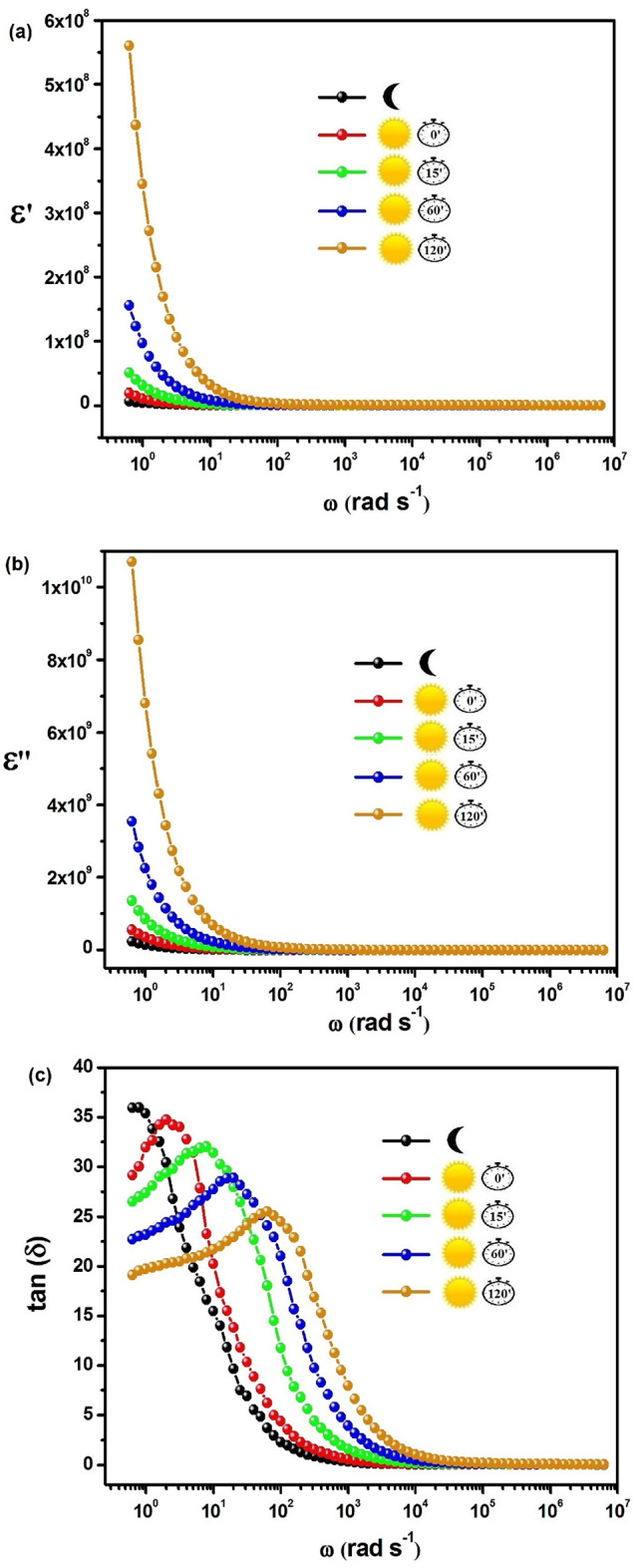
(**a**) Real permittivity (ε’) of Cs₂NaInCl₆ under varying illumination times, (**b**) Imaginary permittivity (ε”), and (**c**) Dielectric loss tangent (tan δ) under different illumination conditions.

As displayed in Fig. 6a–c, the dielectric response of Cs₂NaInCl₆ was studied further under dark and illuminated conditions through both ε′(ω) and ε″(ω), in addition to tan δ(ω). The real permittivity ε′ indicates very large values at a low angular frequency for all illumination times, reaching 10⁷–10⁸ before a steep decline with increasing ω and a tendency towards a nearly constant and much smaller value at high frequencies as seen in Fig. 6a. This behavior characterizes polar materials in which interfacial (Maxwell–Wagner) and space-charge polarization govern at low frequencies, whereas only faster ionic, dipolar and electronic polarization mechanisms dominate at high frequencies^38–40^. It should be pointed out that the intrinsic dielectric constant of the material is not reflected by the very high dielectric permittivity values (10⁷–10⁸) observed at low frequencies, which are primarily associated with extrinsic effects including space-charge accumulation, Maxwell–Wagner interfacial polarization and electrode polarization. The intrinsic dielectric response of Cs₂NaInCl₆ is not only significantly smaller but is also governed by the bulk lattice properties, which is affirmed by the convergence of ε′at high frequencies toward much lower and stable values. Such distinction agrees with the modulus formalism analysis, highlighting the intrinsic bulk relaxation processes and suppressing electrode contributions.

A clear dependence on illumination is noticed. Indeed, ε′ increases systematically at a given low frequency with the illumination time. The highest values were obtained after 120 min of light exposure. This trend confirms that photoexcitation strongly improves the low-frequency polarizability of the material. The rise in ε′ under illumination can be attributed to the generation of long-lived photocarriers which accumulate at grain boundaries and at the electrode–sample interfaces, resulting in pronounced space-charge regions^38^. This is consistent with the impedance analysis. Such behavior evidences that, instead of direct contributions from localized excitonic states, mobile charge carriers and space-charge effects govern the dielectric response. Functioning as additional polarizable entities that follow the applied AC field at low ω, the photo-induced charges lead to a giant effective permittivity. The different curves converge to similar ε′ values at higher frequencies, demonstrating that the intrinsic high-frequency permittivity of the In–Cl network is essentially unaffected by illumination. This behavior is consistent with the structural robustness deduced from XRD measurements. It is also noteworthy that no clearly resolved bleaching feature is observed within the accessible transient spectral range, which further limits the unambiguous assignment of the TAS signal to intrinsic excited-state absorption processes.

Figure 6b displays the imaginary part ε″(ω) which shows a qualitative behavior similar to ε′(ω) with very large values at low frequencies decaying rapidly once ω rises. In the low-frequency region, the large ε″ reveals great dielectric losses associated with space-charge relaxation and conduction processes. As soon as the illumination time increases, ε″ grows strongly at low ω, which proves that the same photo-activated carriers responsible for the improvement of ε′ contribute to dielectric losses through hopping and drift under the applied field^38–40^. The concomitant increase in ε″ and AC conductivity deduced from the impedance measurements indicates the intimate coupling between the dielectric response and charge transport in Cs₂NaInCl₆. ε″ becomes small for all states at high frequencies, demonstrating that, the moment the carriers can no longer follow the rapid field oscillations, conduction-related losses are strongly suppressed.

Figure 6c offers more insight into the loss processes through the evolution of the loss tangent tan δ(ω). Tan δ reveals, at first, a broad maximum in the low-to-intermediate frequency range for each illumination condition, but then, at high frequencies, it notices a gradual decrease reaching low values. As soon as the probing frequency matches the characteristic relaxation time of the dipolar/space-charge entities, the presence of such peaks shows a relaxation process in which the energy dissipation is maximal^38–40^. The position and amplitude of the tan δ maxima are clearly influenced by illumination: the peak slightly shifts to higher angular frequencies while its maximum value drops with growing light exposure^38^. The change to higher frequencies demonstrates a decrease in the characteristic relaxation time, which implies that polarized entities such as localized carriers and interfacial charges reorient or relax faster in the illuminated state. The decline in the peak amplitude proves that, despite the rise in conductivity, the material becomes relatively less lossy, which can be attributed to a more efficient and less hindered charge motion as soon as deep traps are gradually filled.

The non-Debye character of the dielectric relaxation in Cs₂NaInCl₆ is confirmed not only by the broad and asymmetric shape of the tan δ peaks but also by the strong dispersion of ε′ and ε″. This is in complete agreement with the constant phase element description extracted from the equivalent-circuit analysis and with the multi-exponential dynamics revealed by TRPL and TAS. Therefore, the dielectric spectra offer a complementary picture in which illumination activates a broad distribution of relaxation times likely associated with localized excitonic states, photocarriers, trap-state distributions, and interfacial space-charge regions, collectively contributing to the overall polarization and loss behavior of this wide bandgap indium-based double perovskite.

**Fig. 7:**
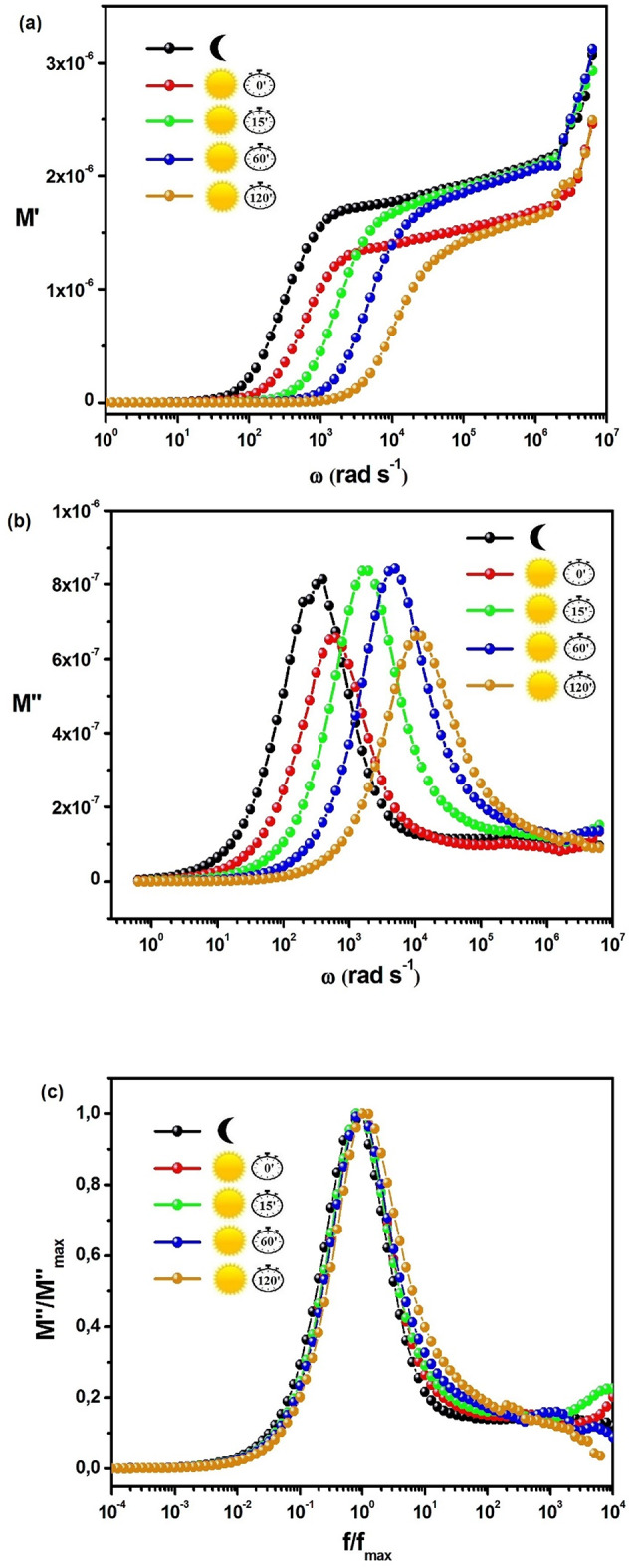
(**a**) Real part of the modulus (M’) as a function of frequency, (**b**) Imaginary part of the modulus (M”), and (**c**) Normalized modulus loss spectra (M”/M”_max_).

The electric modulus formalism was used to further clarify the intrinsic relaxation dynamics of Cs₂NaInCl₆ and to suppress the overwhelming low-frequency contributions which generate from electrode polarization and space-charge accumulation^41^. As shown in Fig. 7a, the frequency dependence of the real part of the modulus, M′(ω), remains close to zero for all illumination conditions at low frequencies. This behavior affirms not only that long-range charge transport is highly screened in this region but also that the response is governed by interfacial polarization, which is in line with the large ε′ values that were previously observed^41^. M′ begins to augment as soon as the frequency increases and eventually indicates a pronounced dispersion in the intermediate-to-high frequency regime. With increasing illumination time, the change in this dispersion towards lower frequencies reveals that the transition from electrode-dominated to bulk-dominated relaxation takes place more readily in the illuminated state. This behavior is consistent with the enhanced population of photoactivated carriers inferred from PL, TRPL and TAS measurements, which collectively suggest that illumination modifies the population and relaxation behavior of localized excitonic states and long-lived photocarriers capable of altering the local relaxation environment. For all illumination conditions, well-defined relaxation peaks are indicated in Fig. 7b by M″(ω), the imaginary part of the modulus. Within the In–Cl framework, localized carriers involved in intrinsic bulk relaxation processes are revealed by these peaks.

The maxima of these peaks is enabled to shift gradually towards lower frequencies through illumination in comparison with the dark state, which implies a rise in the characteristic relaxation time since the Cs₂NaInCl₆ becomes populated with long-lived photocarriers whose dynamics are influenced by trap-state interactions and exciton formation^39–41^.

This observation is qualitatively consistent with the optical signatures of long-lived localized emissive states and trap filling processes, which may contribute to slower local relaxation dynamics compared with shallow-trap or free-carrier relaxation processes. A decline in the overall modulus strength is indicated through the reduction in the amplitude of the M″ peak under illumination. The decrease is in agreement with a more homogeneous relaxation environment that evolves since non-radiative channels are suppressed and deep traps are progressively saturated^39–41^.

Figure 7c shows that the modulus loss spectra were normalized as M″/M″_max_ which was plotted as a function of the reduced frequency f/f_max_ in order to know if illumination can modify the fundamental nature of the relaxation mechanism. Regardless of illumination time, all curves collapse onto a single master curve, proving that, whether the material is in the dark or under illumination, the distribution of relaxation times retains the same shape. This invariance affirms that illumination alters the relaxation rate without fundamentally changing the underlying relaxation mechanism^42^. Such scaling behavior is typical of systems dominated by a broad, temperature-independent and field-independent distribution of localized states^42^. This scenario is consistent with the non-Debye properties inferred from the constant-phase elements in the equivalent-circuit analysis and with the multi-timescale relaxation behavior phenomenologically reflected in the transient optical measurements and more directly supported by the TRPL analysis. Thus, the electric modulus representation provides a clear and coherent picture of the intrinsic short-range carrier dynamics in Cs₂NaInCl₆ and also confirms that the photo-induced processes which are responsible for the enhancement of optical emission and electrical conductivity play a key role in shaping the relaxation behavior of this wide bandgap double perovskite.

**Fig. 8:**
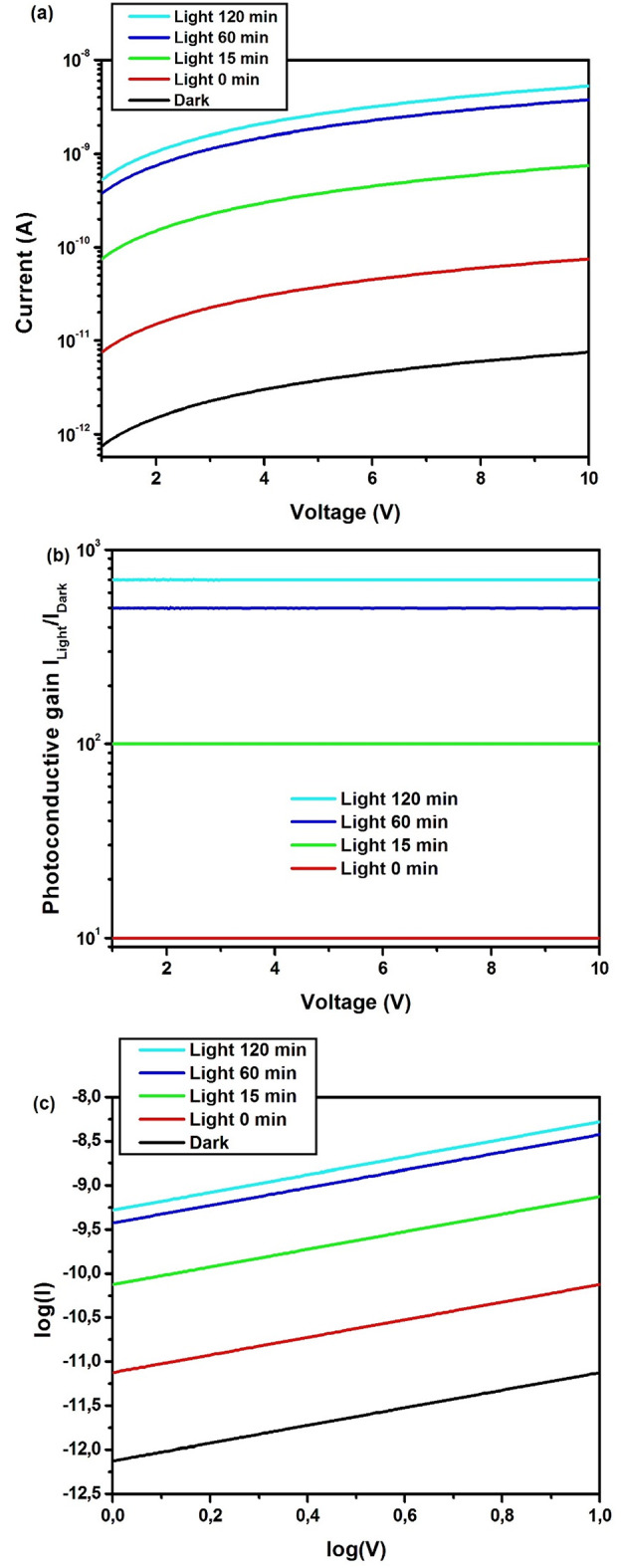
(**a**) Current-voltage (I–V) characteristics of Cs₂NaInCl₆ under dark and illuminated conditions, (**b**) Photoconductive gain (Ilight/Idark) versus illumination time, and (**c**) Double-logarithmic I–V plot showing ohmic transport behavior.

The influence of the photoactivation on charge transport is explained through the I–V characteristics of Cs₂NaInCl₆ measured after various illumination durations and also under dark conditions. Over the investigated voltage range, a linear and stable transport behavior is demonstrated by the current which increases monotonically with the applied bias for all conditions without exhibiting breakdown effects or discontinuities as shown in Fig. 8a. The highly insulating nature of this wide bandgap double perovskite is shown through the current that remains extremely low in the dark. A considerable increase in the current is noticed as soon as the sample is illuminated, knowing that illumination time strongly influences the magnitude of the rise. Reaching its highest values after 120 min, the current continues to grow steadily for longer exposure times after roughly doubling at 0 min of illumination. Promoting more efficient carrier motion within the In–Cl octahedral network and reducing recombination losses, the gradual filling of both shallow and deep trap states and also the growth of the photo-generated carrier population are revealed by this monotonic enhancement^43^.

As displayed in Fig. 8b, the photoconductive gain was estimated as the ratio I_light_/I_dark_ at each voltage in order to quantify the improvement. The conduction mechanism does not change with illumination. This is demonstrated by the gain which remains almost constant with voltage for all applied biases. Nevertheless, with roughly one order of magnitude at 0 min, two orders at 15 min and almost three orders after 60–120 min of light exposure, the magnitude of the gain rises considerably with illumination time. The presence of a persistent photoconductivity regime is affirmed by this strong increase. The persistent photoconductivity is dominated by bulk carrier trapping and detrapping mechanisms instead of interface-controlled processes, which is further indicated by the voltage-independent nature of the photoconductive gain. Even after prolonged exposure, the photo-generated carriers in this regime continue to support conduction and remain trapped in long-lived defect-related states. These changes are in complete agreement with both the impedance measurements which reveal a significant decrease in the bulk resistance R_1_ and the dielectric analysis showing a pronounced enhancement of space-charge polarization at low frequencies. Such interpretation is further supported by the optical measurements, where transient absorption provides qualitative evidence of multi-timescale photoinduced relaxation processes, while PL and TRPL measurements indicate the presence of long-lived localized emissive states together with persistent photoactivated carriers under illumination.

Figure 8c shows that the I–V curves are replotted in double-logarithmic form as log(I) versus log(V) so as to gain additional insight into the nature of the conduction mechanism. All curves exhibit an almost perfect linear relationship with a slope close to unity, which indicates that an ohmic process dominates transport in both dark and illuminated states. The observed ohmic behavior affirms that free carriers govern charge transport. Therefore, instead of any direct contribution from self-trapped excitons, an increased carrier density and trap filling effects lead to the enhancement under illumination. Therefore, illumination increases the absolute value of the current without altering the underlying field dependence of charge motion. The absence of transitions to a super-linear regime excludes space-charge limited conduction or Poole-Frenkel behavior within the explored voltage range. Such observation reveals that the enhancement of current originates primarily from an increased density of mobile carriers rather than from a modification of the conduction mechanism itself. These I–V analyses collectively suggest that illumination activates a significant population of long-lived carriers contributing to a stable and persistent photoconductive response, in agreement with the impedance, dielectric and optical measurements.

**Fig. 9:**
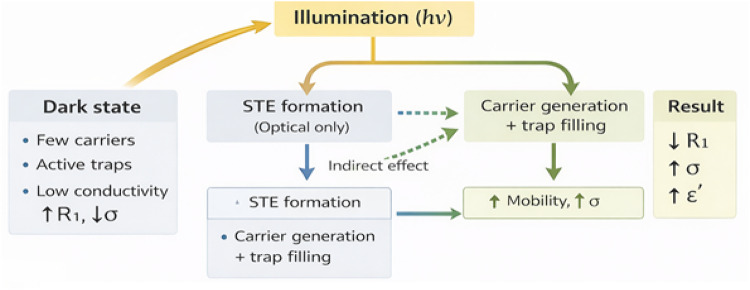
Schematic representation of the photo-activated transport mechanism in Cs₂NaInCl₆ highlighting the indirect influence of self-trapped excitons (STEs) on carrier dynamics.

Figure 9 illustrates a proposed interpretation of the coupled electrical and optical processes the different illumination-activated processes occurring in Cs₂NaInCl₆ in order to consolidate these electrical observations. The material contains only a small population of free carriers and weakly-populated STEs in the dark, which results in high electrical resistance, low permittivity and negligible current. Two parallel photoinduced processes are proposed upon UV excitation: (i) the formation of localized excitonic states dominating the optical response and (ii) trap filling associated with mobile charge carriers governing electrical transport. When illumination continues, long-lived photocarriers accumulate and space-charge regions develop within the material. These effects jointly reduce the bulk resistance, improve the dielectric response and augment both AC and DC conductivity. Thus, the behavior captured in the I–V curves is the macroscopic manifestation of the microscopic processes highlighted by optical spectroscopy and impedance analysis as displayed schematically in Fig. 9.

Altogether, under illumination, a consistent physical interpretation of the behavior of Cs₂NaInCl₆ is revealed by the combined electrical, optical and structural analyses. The formation of long-lived localized states and lattice relaxation follows optical excitation and is consistent with the emergence of localized excitonic states and subsequent relaxation processes within the In–Cl octahedral framework. Enhanced radiative emission and extended exciton lifetimes result from such species which lessen non-radiative pathways and gradually fill defect-related traps. Persistent photoconductivity, reduced charge-transfer resistance and increased dielectric polarization are caused by a fraction of the photo-excited carriers which simultaneously becomes mobile over longer timescales. Therefore, the DC I–V characteristics, AC electrical behavior, relaxation dynamics and optical signatures observed across all measurements are explained by the interplay of localized excitonic processes, trap-state modulation and mobile carrier dynamics. In this wide bandgap indium-based double perovskite, the intrinsic coupling between charge-transport processes and excitonic physics is suggested by the overall behavior.

## Conclusion and outlook

The thermally-stable and cubic framework, low structural disorder and wide bandgap of Cs₂NaInCl₆ constitute a great foundation for advanced electrical and optical functionalities, which is why it has emerged as an intrinsically excitonic and structurally robust lead-free double perovskite. This work suggests that optical excitation in Cs₂NaInCl₆ is accompanied by multi-timescale relaxation processes associated with localized excitonic emission and photoactivated carrier dynamics. While the optical response is predominantly consistent with STE-mediated recombination, contributions from defect-related localized states cannot be completely excluded. The TAS measurements provide only qualitative insight into short-timescale photoinduced dynamics due to the spectral overlap with the PL emission band and the limited temporal resolution of the experiment. Consequently, the transient results are interpreted cautiously and are not used as definitive proof of intrinsic excited-state absorption or direct ultrafast STE formation. Minor contributions from defect-related states cannot be completely ruled out although the emission is predominantly attributed to STE recombination. As shown through pronounced persistent photoconductivity, improved dielectric polarization and a strong reduction of the bulk resistance, complementary electrical characterizations indicate that, while maintaining an underlying ohmic transport regime, illumination considerably enhances charge transport through the activation of long-lived photocarriers and trap filling processes. Therefore, although contributions from metastable defect configurations cannot be entirely excluded, long-lived carrier trapping in bulk defect states leads to the observed persistent photoconductivity. Photo-generated carriers primarily govern charge transport while the optical response is dominated by STEs. The indirect influence of localized excitonic processes on carrier trapping and recombination dynamics is likely associated with the observed coupling between the electrical and optical properties. All these results allow Cs₂NaInCl₆ to be a model system for intrinsically-excitonic halide double perovskites by suggesting a consistent microscopic–macroscopic interpretation which joins exciton dynamics with AC and DC transport behavior. Beyond existing literature, this combined optical–electrical approach offers new insight by establishing a physically-supported connection between the macroscopic transport behavior and exciton dynamics. Multiple promising opportunities for future applications are opened up owing to the optoelectronic behavior of the material uncovered in this work added to its fundamental interest. The potential of Cs₂NaInCl₆ for deep-blue and ultraviolet photonic devices is emphasized by the efficiency and stability of the STE-mediated blue emission. The suitability for capacitive architectures and high-frequency dielectric components is provided through the non-Debye relaxation behavior and the strong dielectric response. In addition, this material can serve as a platform for ionizing not only scintillation-based detection technologies in which fast exciton formation and long-lived carrier states are fundamental but also radiation sensors and UV photodetectors as indicated by long-lived photo-activated carriers and persistent photoconductivity. Moreover, a roadmap for rational compositional engineering in A₂B⁺B³⁺X₆ systems is offered by the intimate coupling of transport properties, lattice relaxation and exciton self-trapping. This may help guide the future design of new lead-free double perovskites with tailored photoconductive, excitonic or dielectric responses.

Altogether, this study builds a solid foundation for device-level exploration and future optimization strategies. It also makes it easier to understand the basic transport and photo-physical mechanisms of Cs₂NaInCl₆. The technological potential of indium-based double perovskites in environmentally-friendly and next-generation applications of photonics and optoelectronics will likely be unlocked by thin-film fabrication, interface engineering, aliovalent substitution, etc. to a larger degree. It is noteworthy, however, that the further advanced spectroscopic or theoretical investigations are required for a fully quantitative description and that the assignment of specific relaxation times to individual microscopic processes remains indicative. A quantitative assignment of the ultrafast transient dynamics would require additional wavelength-dependent transient absorption measurements, global kinetic analysis, and experimental configurations specifically designed to suppress possible PL-related contributions. Further theoretical and time-resolved investigations would be required to establish a causal microscopic mechanism even though the present results provide strong experimental correlations.

## Supplementary Information

Supplementary Material 1

## Data Availability

The datasets generated and/or analyzed during the current study are available in the Zenodo repository, https://doi.org/10.5281/zenodo.18992536.
