# Supplementary material for: Photo-activated charge transport and exciton dynamics in Cs₂NaInCl₆ double perovskite
Source: Sci Rep. 2026 Jun 4;16:25613. doi: 10.1038/s41598-026-55947-2 (PMC13478166; doi:10.1038/s41598-026-55947-2)
Supplement: Supplementary file 1 [file 41598_2026_55947_MOESM1_ESM.docx]

**Supporting Information**

**Photo-Activated Charge Transport and Exciton Dynamics in Cs₂NaInCl₆ Double Perovskite**

Fahad K. Alshammari ^1^, Abdullah A. Alatawi ^2^, Achref Jebnouni ^3^, Amjad Salamah Aljaloud ^1^, Turki Alkathiri ^4, *^, Ahmed F Almutairi ^5^, Bandar F. Alsaleh ^1^, Mohamed Bouzidi ^1^, Mohamed Ben Bechir ^6, 7 *^

*^1^ Department of Physics, College of Science, University of Ha'il, Ha'il P.O. Box 2440, Saudi Arabia*

*^2^ King Abdulaziz City for Science and Technology, P.O. Box 6086, 11442 Riyadh, Saudi Arabia*

*^3^ Medical Surgical Nursing Department, College of Nursing, University of Ha’il, Ha’il, Saudi Arabia*

*^4^ Department of Electrical Engineering, College of Engineering, Al-Baha University, P.O. Box 65799, Albaha, Saudi Arabia*

*^5^ Department of Mechanical Engineering, College of Engineering at Yanbu, Taibah University, Yanbu Al-Bahr 41911, Saudi Arabia*

***^6^*** *Laboratory of Spectroscopic and Optical Characterization of Materials (LaSCOM), Faculty of Sciences, University of Sfax, BP1171 – 3000 Sfax, Tunisia*

***^7^*** *Department of Physics, Faculty of Sciences of Gafsa, University of Gafsa, 2112, Tunisia*

*^*^* *Corresponding author. E-mail address:* [*talkathiri@bu.edu.sa*](mailto:talkathiri@bu.edu.sa)*,* [*mohamedbenbechir@hotmail.com*](mailto:mohamedbenbechir@hotmail.com)

**Supplementary Figures**

**Fig. S1.** X-ray diffraction refinement profile for Cs₂NaInCl₆, showing good agreement between experimental and simulated data.

**Fig. S2.** (a) Transient absorption map ΔA(λ,t), (b–d) Kinetic traces of transient absorption at 445 nm measured at different temperatures (300 K, 150 K, and 80 K), and (e) Spectral evolution at selected time delays.

**Fig. S3.** Equivalent circuit model fitted to the impedance spectra of Cs₂NaInCl₆, showing the contribution of space-charge polarization and bulk relaxation processes.

**Supplementary Tables**

**Table S1.** Fitted parameters for transient absorption kinetic traces at various temperatures, including decay times and relaxation processes.

| T (K) | A₁ | τ₁ (ps) | A_2_ | τ₂ (ps) | A_3_ | τ₃ (ps) | C | χ² | R² |
| --- | --- | --- | --- | --- | --- | --- | --- | --- | --- |
| 300 | 0.4888 ± 0.0459 | 29.7 ± 2.6 | 0.3680 ± 0.0371 | 155 ± 28 | 0.1450 ± 0.0212 | 1473 ± 352 | 0.0065 ± 0.0059 | 0.00049 | 0.9968 |
| 150 | 0.4797 ± 0.0529 | 40.4 ± 3.4 | 0.3074 ± 0.0394 | 178 ± 43 | 0.2000 ± 0.0292 | 1179 ± 197 | 0.0189 ± 0.0040 | 0.00029 | 0.9981 |
| 80 | 0.5051 ± 0.0217 | 50.7 ± 2.3 | 0.3439 ± 0.0192 | 335 ± 44 | 0.1462 ± 0.0184 | 2630 ± 677 | 0.0160 ± 0.0079 | 0.00021 | 0.9986 |

**Supplementary Equations**

$y=A_{1}e^{{-x}/{\tau_{1}}}+A_{2}e^{{-x}/{\tau_{2}}}+A_{3}e^{{-x}/{\tau_{3}}}+C$	(S1)

where A_1_, A_2_, and A_3_ are amplitudes, τ_1_, τ_2_, and τ_3_ are the decay times, and C is a constant offset.
